# Novel Compound Heterozygous Mutations in *IL-7 Receptor* α Gene in a 15-Month-Old Girl Presenting With Thrombocytopenia, Normal T Cell Count and Maternal Engraftment

**DOI:** 10.3389/fimmu.2019.02471

**Published:** 2019-10-30

**Authors:** Paola Zangari, Cristina Cifaldi, Silvia Di Cesare, Gigliola Di Matteo, Maria Chiriaco, Donato Amodio, Nicola Cotugno, Maia De Luca, Cecilia Surace, Saverio Ladogana, Simone Gardini, Pietro Merli, Mattia Algeri, Paolo Rossi, Paolo Palma, Caterina Cancrini, Andrea Finocchi

**Affiliations:** ^1^Unit of Immune and Infectious Diseases, Academic Department of Pediatrics, Bambino Gesù Children's Hospital, IRCCS, Rome, Italy; ^2^Department of Systems Medicine, University of Rome Tor Vergata, Rome, Italy; ^3^Research Unit in Congenital and Perinatal Infections, Unit of Immune and Infectious Diseases, Academic Department of Pediatrics, Ospedale Pediatrico Bambino Gesù, IRCCS, Rome, Italy; ^4^Laboratory of Medical Genetics Unit, Bambino Gesù Children's Hospital, IRCCS, Rome, Italy; ^5^Paediatric Onco-Haematology Unit, Casa Sollievo della Sofferenza Hospital, IRCCS, San Giovanni Rotondo, Italy; ^6^GenomeUp SRL, Rome, Italy; ^7^Department of Pediatric Hematology and Oncology, Bambino Gesù Children's Hospital, IRCCS, Rome, Italy

**Keywords:** SCID, *IL-7R*α gene, novel compound heterozygous mutations, atypical onset, maternal engraftment

## Abstract

Patients with severe combined immunodeficiency (SCID) exhibit T lymphopenia and profound impairments in cellular and humoral immunity. IL-7 receptor α (IL-7Rα) deficiency is a rare form of SCID that usually presents in the first months of life with severe and opportunistic infections, failure to thrive and high risk of mortality unless treated. Here, we reported an atypical and delayed onset of IL7Rα-SCID in a 15-month-old girl presenting with thrombocytopenia. Immunological investigations showed a normal lymphocyte count with isolated CD4-penia, absence of naïve T cells, marked hypergammaglobulinemia, and maternal T cell engraftment. Targeted next generation sequencing (NGS) revealed two novel compound heterozygous mutations in the *IL-7R*α gene: c.160T>C (p.S54P) and c.245G>T (p.C82F). The atypical onset and the unusual immunological phenotype expressed by our patient highlights the diagnostic challenge in the field of primary immunodeficiencies (PID) and in particular in SCID patients where prompt diagnosis and therapy greatly affects survival.

## Highlights

- Normal T cell count with isolated CD4-penia and thrombocytopenia in a SCID patient.
- Novel compound heterozygous mutations in IL-7 receptor α deficiency.
- Maternal T cell engraftment.

## Background

Severe combined immunodeficiency (SCID) represents a heterogeneous group of primary immunodeficiencies with a severe impairment of T cell function and variable functional or quantitative B and natural killer (NK) cell defects, leading to severe and opportunistic infections within the first months of life (1). Interleukin-7 (IL-7) is essential for T cell development in the thymus and for maintaining and restoring the homeostasis of mature T cells, but it is redundant for human B and NK cell development. The IL-7 receptor (IL-7R) is a heterodimer, consisting of two subunits, the IL-7Rα chain (CD127) and common-γ chain (CD132). The binding of IL-7 to the IL-7Rα chain leads to dimerization with the common-γ chain and a subsequent activation of the Janus kinase–signal transducer and activator of the transcription (JAK–STAT) pathway (2). *IL-7R*α mutation is responsible of about 10% of SCID and generally causes an early onset SCID with a T- B+ NK+ immunophenotype (3). Besides typical characteristics of the SCID phenotype, *IL-7R*α mutations could manifest later with a milder clinical presentation and rarely with immune dysregulation such as autoimmune cytopenia or Omenn syndrome (OS) (4–6). Moreover, patients with SCID do not usually recognize and reject foreign cells, and maternal engrafted T cells were therefore detected in up to 40% of patients (7). In the majority of these cases, maternal engraftment is asymptomatic, however some infants can manifest signs and symptoms of graft-versus-host-disease (GVHD) with cutaneous involvement, liver injury, and hematologic abnormalities. Here, we report a 15-month-old girl with an atypical presentation of thrombocytopenia, isolated CD4-penia, hypergammaglobulinemia and maternal T-cell engraftment who was found to have two novel compound heterozygous mutations in *IL-7R*α.

## Case Presentation

A 15-month-old Caucasian girl born from non-consanguineous parents with an uneventful dizygotic twin pregnancy, good birth weight, and healthy twin sister was referred to our hospital due to mild atopic dermatitis and recurrent upper respiratory infections.

There was no known family history of immunodeficiency, the baby had normal psychomotor development and there was no failure to thrive.

At 10 months of age she experienced periorbital edema due to primary EBV infection as confirmed by blood EBV PCR and subsequent hypertransaminasemia. At 12 months of age she was hospitalized for immune thrombocytopenic purpura, which was successfully treated with high-dose intravenous immunoglobulin (IVIG).

At the time of our evaluation, immunological investigations revealed a normal count of total lymphocytes (4,300 cells/mm^3^), severely reduced numbers of CD3^+^CD4^+^ (184 cells/mm^3^) with barely detectable CD4^+^ CD45RA^+^ CD27+ naïve T cells and CD4^+^ CD45RA^+^ CD31+ recent thymic emigrants (RTE). *In vitro* T-cell proliferation in response to phytohemagglutinin (PHA) and anti-OKT3 was severely decreased. Despite her marked hypergammaglobulinemia, partially impaired humoral immunity with reduced pneumococcal IgG titer (total pneumococcal IgG ELISA Binding Site) after vaccinations (Prevnar13, two doses), lower peripheral B cells with reduced transitional B cells and increased plasma cells were found. Interestingly, protective antibody titers to tetanus and to Haemophilus influenzae type B (Hib) immunization were detected (Table 1).

**Table 1:** Patient's immunological investigations.

	**Patient value**	**Normal age-matched value**
**COMPLETE BLOOD COUNT**		
Complete blood count		
White blood cell (103/μl)	10.41	6.00–17.00
Red blood cells (106/μl)	4.27	3.60–5.00
Neutrophils (103/μl)	3.47	1.68–8.50
Lymphocytes (103/μl)	4.38	3.6–8.9
Platelets (103/μl)	371	150–450
Hemoglobin (g/dl)	11	10.5–15.5
**LYMPHOCYTE SUBSET**		
Absolute CD3+	2400	2,100–6,200
Absolute CD4+	184	1,300–3,400
Absolute CD8+	2012	620–2,000
Absolute CD16+CD56+	1505	720–2,600
Absolute CD19+ cells/mL	283	720–2,600
CD19+CD24+CD38+ cells/mL (Transitional B)	18	56–101
CD19+CD24-CD38+ cells/mL (Plasma cells)	70	40–55
CD3+CD4+CD27+CD45RA+% (naÏve)	0.3	63–91
CD3+CD4+CD27+CD45RA-% (CM)	66.3	10–26
CD3+CD4+CD27-CD45RA-% (EM)	33.4	3–16
CD3+CD4+C27-CD45RA+% (TEMRA)	0.1	3–12
CD3+CD4+CD31+CD45RA+% (RTE)	0.2	>40
CD3+CD8+CCR7+CD45RA+ % (naÏve)	0.07	71–98
CD3+CD8+CCR7+CD45RA-% (CM)	0.24	3–15
CD3+CD8+CCR7-CD45RA-% (EM)	97.9	9–47
CD3+CD8+CCR7-CD45RA+% (TEMRA)	1.7	7–25
CD3+ TCR alpha-beta	96	26–100
CD3+ TCR gamma-delta	3.6	1–13
**LYMPHOCYTE STIMULATION TO MITOGENS (cpm)**		
Phytohemagglutinin	2.788	>35.000
OKT3	4.838	>25.000
**SERUM IMMUNOGLOBULIN LEVELS**		
IgG (mg/dL)	3,603	264–1,509
IgA (mg/dL)	680	17–178
IgM (mg/dL)	509	48–337
IgE (IU/mL)	2.9	<2
**ANTIBODY RESPONSE TO VACCINATIONS**		
Tetanus titer (IU/mL)	0.13	>0.1
Haemophilus titer (mg/l)	0.2	>0.1
Post-pneumococcal titer (mg/l)	<3	>35

*CM, central memory; EM, effector memory; TEMRA, terminally differentiated effector memory; RTE, recent thymic emigrants*.

Microbiological tests revealed negative HIV serology and incomplete seroconversion against EBV (IgG anti-VCA present, IgM anti-VCA absent, IgG anti-EBNA absent) with a high level of EBV replication.

Chest CT showed hypoplasic thymus, multiple bilateral nodular lesions with ground glass appearance and 6,000,000 copies/mL of EBV in bronchoalveolar lavage. No other significant pathogens were identified. An abdomen ultrasound documented hepatomegaly and blood tests revealed hypertransaminasemia with normal markers of liver synthetic function.

Moreover, due to the persistence of eczematous dermatitis, a skin biopsy was performed that revealed psoriasiform epidermal hyperplasia, focal spongiosis, and parakeratosis and an inflammatory mononuclear dermal infiltrate with the following immunohistochemical profile: CD20^−^; CD3^+^; CD8^+/−^; CD4^−/+^; CD68^−/+^; CD1a^+^.

In view of the marked CD4-penia with almost absent recent RTE, the concomitant expansion of the CD4^+^ and CD8^+^memory T lymphocytes and the cutaneous histopathology a diagnosis of SCID with maternal T-cell engraftment was suspected. The variable number of tandem repeat analysis performed on both peripheral blood and the skin biopsy revealed that all T lymphocytes (CD4^+^ and CD8^+^) in the patient were of maternal origin while CD19^+^ and CD16^+^CD56^+^ cells were autologous (Supplementary Figure 2).

**Figure 1:**
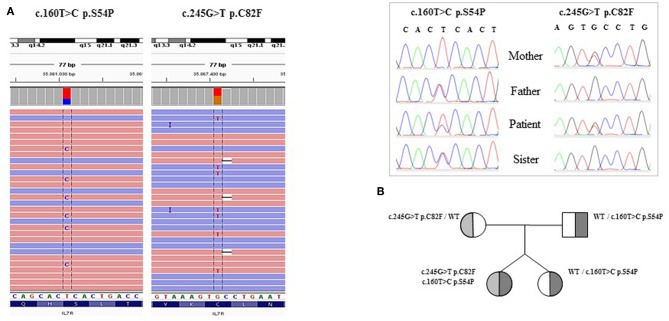
**(A)** Sequence alignment of the patient's BAM file on IGV (Integrative Genomics Viewer) and the two heterozygous mutation found by *IL7R* DNA sequencing. **(B)** Patient's genealogical tree.

Targeted NGS (Ion Torrent), revealed two novel compound heterozygous mutations in *IL-7R*α gene confirmed by Sanger sequencing (Figure 1A). The c.160T>C (p.S54P) was predicted to be damaging by SIFT and PROVEAN, probably damaging by Polyphen whereas CADD assigned a score of 18, predicting this variant as likely benign. The second mutation, c.245G>T (p.C82F), is predicted to be damaging by all pathogenicity prediction tools. Phylogenetic and amino acid conservation analyses, when available, showed that the serine at position 54 was conserved in many amniota vertebrates but changed to leucine in pig, dog, marmot, and other species, whereas the cysteine at position 82 was well-conserved.

To investigate the potential consequences of these mutations to the protein stability and structure we analyzed the 3D-structure of Interleukin-7 receptor subunit alpha (IL7R—UniProtKB:P16871) (8). The X-ray-resolved structure of IL7R was retrieved from the Protein Data Bank with the PDB ID code 3UP1 (9). The structural features of the IL7R were analyzed with Open source PyMOL v. 1.7.1.0. (10). The C82 was involved in disulfide bond formation with C74 (Supplementary Figure 1A) and the S54 was involved in moderate electrostatic bond formation with Q45 (hydrogen bond distance 2.7A, Supplementary Figure 1B), both destabilizing the IL7R antiparallel beta-sheets.

For the protein stability prediction, we performed the mCSM tool (11), and the predicted stability change (ΔΔG) was −0.895 Kcal/mol (Destabilizing) and −0.816 Kcal/mol (Destabilizing), respectively.

The pedigree analysis indicated the presence of the p.C82F mutation in the mother, and the p.S54P mutation in the father and the proband's sister (Figure 1B).

**Figure 2:**
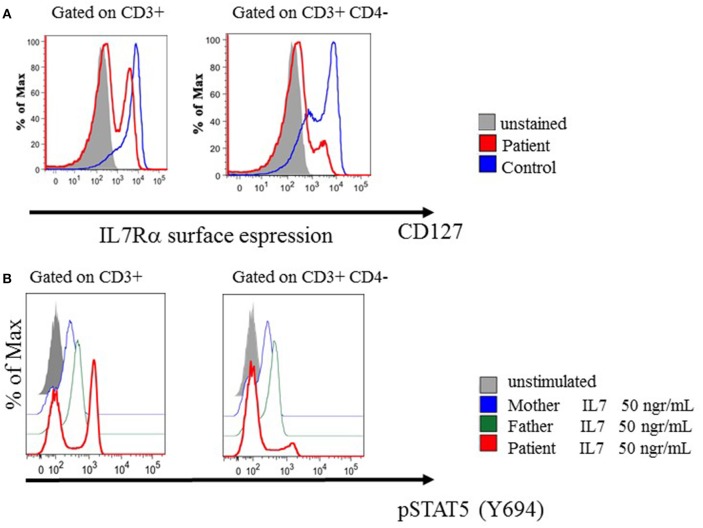
Phenotypic and pSTAT5 signaling in patient's relatives and in patient derived lymphocytes. **(A)** IL-7Rα (CD 127) surface expression were assessed by means of multiparameter flow cytometry in T cell subset. **(B)** The phosphorylation status of STAT5 (Y694) was determined by means of Phosflow analysis after IL-7 treatment in T cell subsets.

The IL-7Rα expression was reduced in total CD3^+^ T cells with a bimodal pattern explained by normal expression on CD3^+^CD4^+^cells (data not showed) and marked reduction on CD3^+^CD4^−^ T cells (Figure 2). In line with these results, STAT5 phosphorylation in response to IL-7 stimulation (50 ng/mL) was reduced in total CD3^+^, normal in CD3^+^CD4^+^cells and almost absent in CD3^+^CD4^−^ T lymphocytes compared to her parents (Figure 2). Regular intravenous immunoglobulin and prophylactic antimicrobial therapy were administered from the time of the diagnosis.

She received a short course of intravenous corticosteroids (three doses of methylprednisolone 15 mg/kg) with a rapid improvement of the dermatitis.

In order to control the EBV replication she received four doses of Rituximab (375 mg/m^2^) with viremia reduction and a full recovery in lung parenchyma was confirmed with a subsequent chest CT.

The patient underwent hematopoietic stem cell transplantation (HSCT) from an HLA-haploidentical donor (her mother) after negative depletion of Tαβ+/CD19+ lymphocytes. The conditioning regimen included treosulfan, fludarabine, anti-T-lymphocyte globulin, and rituximab; no post-transplant pharmacological graft-versus-host disease (GVHD) prophylaxis was employed. The median time to reach neutrophil and platelet engraftment was 31 and 11 days, respectively. No toxicities or serious adverse events occurred. A total of 35 months after the allograft she is in good clinical conditions and without GVHD.

## Discussion

IL-7Rα deficiency represents ~10% of SCID cases. IL-7 signaling is essential for normal T-cell development leading to the activation of the phosphatidylinositol-3-kinase (PI3K), the Ras/Raf signaling cascade and Janus kinase/STAT pathways (STAT5 and STAT3) (2). Typically, patients with complete IL-7Rα deficiency present early onset SCID with profound T cell lymphopenia and abnormal T-cell function. B-cell levels are typically normal, but immunoglobulin production is usually decreased due to T cell dysfunction. Our patient showed an atypical IL-7Rα-deficient SCID phenotype, including later onset, isolated CD4-penia with a normal T cell count and hypergammaglobulinemia. Profound or moderate T cell lymphopenia have been reported in all patients affected by IL-7Rα deficiency (12–14). In our case the immunological features suggesting a combined immunodeficiency were the severely reduced number of circulating CD4+ T lymphocytes, the naïve CD4+ T cells absence, the defective T-cell proliferation and the abnormal peripheral B-cell subset distribution. Of note was the fact that the absolute lymphocyte count and γδ T cells were normal.

Using NGS techniques we detected the presence of two probable pathogenic novel compound mutations, the p.S54P and the p.C82F both destabilizing the IL7R protein structure. Pathogenicity of the cysteine (C82F) and the serine (S54P) replacements can be attributed to the structural role of these amino acids. Cysteine is often involved in disulphide bonds, where these bonds stabilize the protein structure. The structure of many extracellular proteins is stabilized by the topology of multiple disulphide bonds. IL7R have three disulfide bonds and one extracellular region. The serine 54 was found in a common motif of the secondary structure, the beta-sheet. Proline can often be found in the secondary structures as there is a turn when the chain must modify its direction. We have therefore hypothesized that these two mutations have a destabilizing and damaging effect. The deleterious effect of these mutations is supported by the maternal engraftment observed in our patient, underlining a compromised immune system that is probably due to an impaired IL7Rα function. Moreover, we had previously ruled out the presence of mutations in 40 genes known to be associated with SCID-CID phenotypes included in the targeted NGS panel used in this study. However, we can't exclude the presence of mutations in other genes not included in the analysis. The maternal engraftment could have had an impact on the atypical phenotype and definitively on the circulating lymphocyte count. Maternal engraftment occurred in ~40% of patients with T- B+ NK- SCID. Notably, a variable frequency in different studies has been previously reported in IL-7Rα-deficient patients (7, 12). The engrafted T cells are usually oligoclonal memory/effector cells, which are non-functional based on *in vitro* studies (7, 12, 15). In line with this, on the circulating T cells, which are almost exclusively of maternal origin, the expression of IL7Rα showed a bimodal pattern. This is probably due to the downregulation of CD127 on CD8+ T cells as they progress through the effector stages (16) and explains the observed impaired IL-7 signaling of the CD3+CD4- subset (Figure 2). However, these cells could provide some degree of immune response, protecting from severe infections and, in some infants, leading to GVHD with cutaneous involvement (rashes, erythrodermia, alopecia), liver injury involvement (hepatosplenomegaly with elevated liver enzymes, cholestasis) and hematologic abnormalities such as eosinophilia, thrombocytopenia and hemophagocytosis. Interestingly, it has been reported in SCID patients that an attenuated or absent alloreactive response *in vivo* correlates with a preponderance of circulating maternal CD8+ T cells, whereas the risk and severity of GVHD is associated with circulating maternal CD4+ T cells (17). Our patient, who almost exclusively had circulating maternal CD8+ T cells, showed clinical and histological evidence of mild GVHD that completely resolved after a short course of corticosteroid therapy.

Moreover, our patient showed a hypergammaglobulinemia with normal antibody titers to some vaccine antigens. Hypergammaglobulinaemia was also reported in two other children with IL-7Rα-deficient SCID and maternal engraftment (13, 14). Considering the age of our patient it is unlikely that these levels could reflect the presence of maternally derived IgG, but it could rather be a hallmark of this atypical phenotype. Moreover, in line with previous reports describing a leaky phenotype due to IL-7Rα hypomorphic mutations (4, 5), our patient suffered from clinical features typically suggestive of immunedysregulation (such as thrombocytopenia and hypergammaglobulinemia), probably due to defective tolerance mechanisms (18).

The diagnosis of SCID is still a challenge in patients with atypical onset and an unusual clinical and immunological phenotype. Our report highlights the atypical clinical presentation of SCID due to two novel compound mutations of *IL7R*α. In the present case, a combination of self-tolerance perturbation, expressed as maternal engraftment and autoimmune manifestations, delayed the SCID diagnosis. Such a clinical scenario, characterized by early onset autoimmune diseases, isolated CD4-penia despite normal total T cell count and hypergammaglobulinemia, should guide physicians toward proceeding to an NGS approach in order to identify pathogenic variants of atypical PID (19).

## Concluding Remarks

In conclusion, this report describes an atypical clinical and immunological presentation of IL-7Rα deficiency. It is important to alert clinicians to consider a diagnosis of IL-7Rα deficiency even in patients with a normal T-cell count and an absence of classical clinical presentation of SCID. Patients with SCID might lack the functional immunity required to reject circulating maternal T cells, resulting in persistent maternal engraftment in up to 40% of patients. Since a diagnostic delay in these patients may be fatal, clinicians should be aware that immune dysregulation manifestations and maternal engraftment should lead to an investigation for possible PID. In this context, NGS techniques play an important role in addressing the diagnosis.

## Data Availability Statement

All relevant datasets for this study are included in the article/Supplementary Material.

## Ethics Statement

All procedures performed in the study were in accordance with the ethical standards of the institutional research committee and with the 1964 Helsinki declaration. Written informed consent, following standard ethical procedures with approval of the Children's Hospital Bambino Gesù Ethical Committee, was obtained from the parents of the patient for the publication of the case report and any potentially-identifying information.

## Author Contributions

PZ and AF wrote the manuscript. CCi, SD, GD, MC, CS, and SG performed the experiments and analyzed the data. CCi, SD, GD, PR, PP, SG, and CCa contributed to the data interpretation. PZ, DA, NC, MD, SL, PM, MA, PR, PP, CCa, and AF followed up on the patient. All authors reviewed and approved the manuscript.

### Conflict of Interest

SG is CEO of the company GenomeUp SRL. The remaining authors declare that the research was conducted in the absence of any commercial or financial relationships that could be construed as a potential conflict of interest.
