# Supplementary figures and images for: Novel Compound Heterozygous Mutations in IL-7 Receptor α Gene in a 15-Month-Old Girl Presenting With Thrombocytopenia, Normal T Cell Count and Maternal Engraftment
Source: Front Immunol. 2019 Oct 30;10:2471. doi: 10.3389/fimmu.2019.02471 (PMC6831519; doi:10.3389/fimmu.2019.02471)

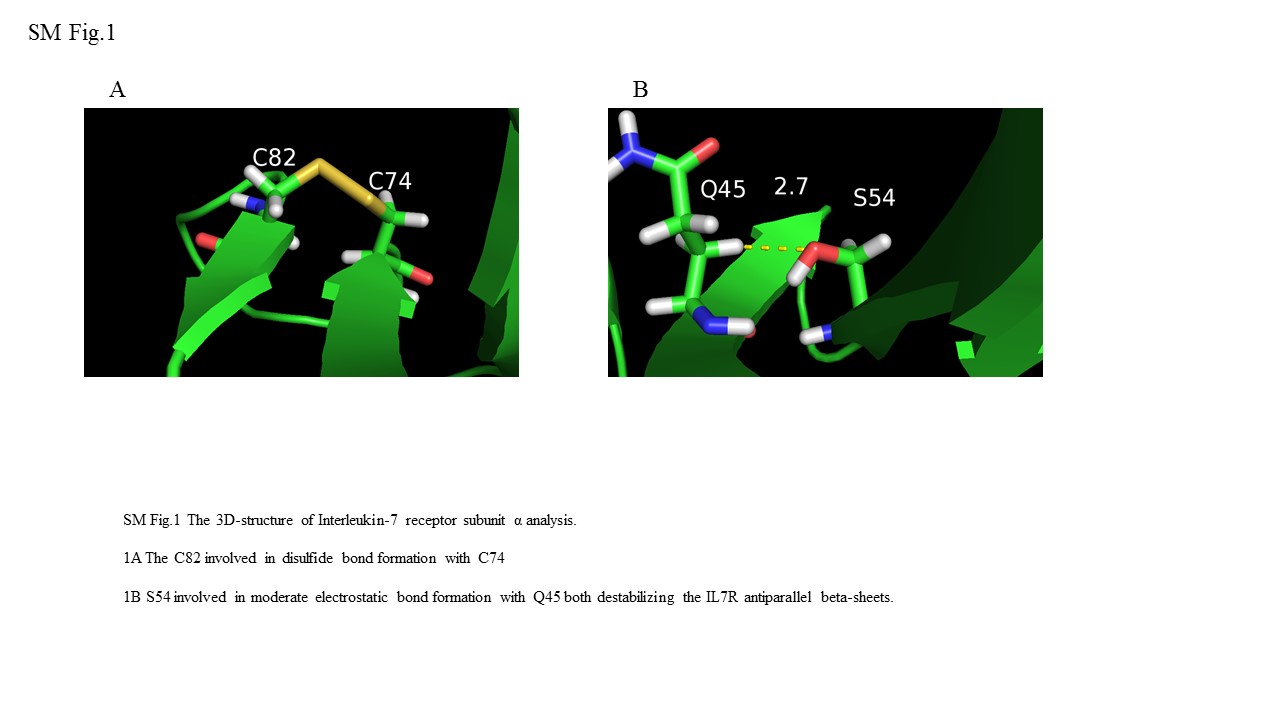

Supplement: Supplementary file 1 [file Image_1.JPEG]

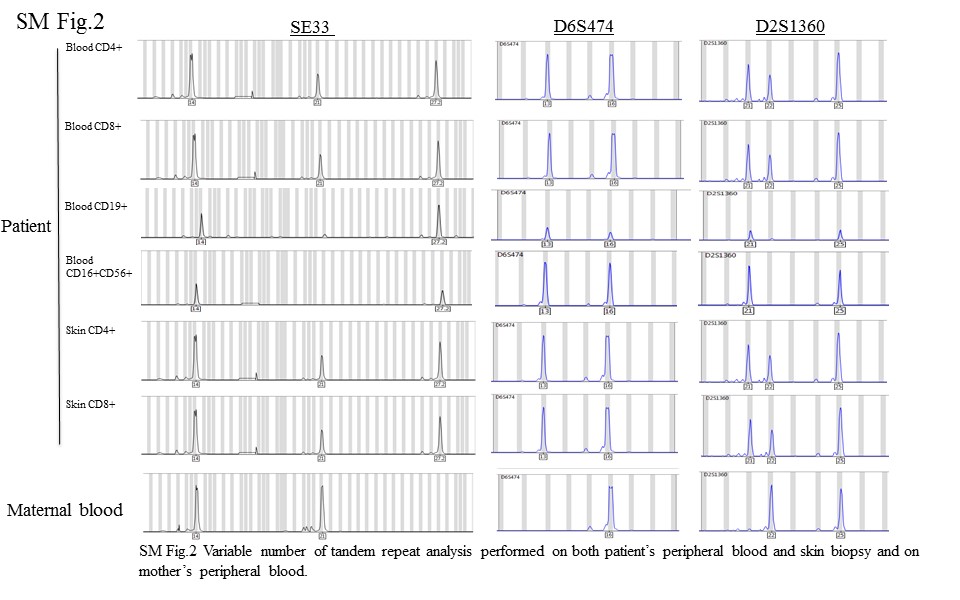

Supplement: Supplementary file 2 [file Image_2.JPEG]
